# Repression of the expression of proinflammatory genes by mitochondrial transcription factor A is linked to its alternative splicing regulation in human lung epithelial cells

**DOI:** 10.1186/s12865-021-00464-2

**Published:** 2021-12-07

**Authors:** Jinsong Luo, Hong Liu, Daniel K. Jun Li, Bin Song, Yi Zhang

**Affiliations:** 1 Department of Pediatrics, Renmin Hospital of Wuhan University, Wuhan, Hubei China; 2 ABLife BioBigData Institute, Wuhan, Hubei China; 3 Department of Biology and Biotechnology, School of Chemistry, Chemical Engineering and Life Science, Wuhan University of Technology, Wuhan, Hubei China

**Keywords:** TFAM, A549, Viral response pathway gene expression, Transcription factors, Alternative splicing

## Abstract

**Background:**

Mitochondrial transcription factor A (TFAM) is associated with a number of neurodegenerative diseases and also with asthma. TFAM deficiency-induced mitochondrial DNA stress primes the antiviral innate immune response in mouse embryonic fibroblasts. However, the role of TFAM in asthma related inflammation remains obscure. The purpose of this study was to investigate the regulatory mechanism of TFAM in asthma.

**Results:**

In this study, we overexpressed TFAM in human lung epithelial cells (A549), then obtained the TFAM-regulated transcriptome by Illumina sequencing technology. Transcriptome analysis revealed that TFAM overexpression down-regulated and up-regulated the expression of 642 and 169 differentially expressed genes (DEGs), respectively. The TFAM-repressed genes were strongly enriched in cytokine-mediated signaling pathway, type I interferon- and INF-γ-mediated signaling pathways, and viral response pathways. We also revealed that 2563 alternative splicing events in 1796 alternative splicing genes (ASGs) were de-regulated upon TFAM overexpression. These TFAM-responding ASGs were enriched in DNA repair, nerve growth factor receptor signaling pathway, and also transcription regulation. Further analysis revealed that the promoters of TFAM-repressed DEGs were enriched by DNA binding motifs of transcription factors whose alternative splicing was regulated by TFAM.

**Conclusions:**

These findings suggest that TFAM regulates not only immune response gene expression in human lung epithelial cells, but also pre-mRNA alternative splicing which may mediate transcriptional regulation; this TFAM-centered gene regulation network could be targeted in developing therapies against various diseases.

**Supplementary Information:**

The online version contains supplementary material available at 10.1186/s12865-021-00464-2.

## Background

Mitochondria are critical in maintaining cellular homeostasis by housing the oxidative phosphorylation machinery for aerobic adenosine triphosphate (ATP) production and several metabolic pathways. In fact, mitochondria are responsible for 90% of cellular energy generation and therefore are heavily involved in sensing and responding the stressful conditions such as inflammation by cells and bodies [1]. In fact, mitochondrial DNA are emerging as a powerful proinflammatory ligand that can be released into cytosol or extracellular environment to stimulate various pattern-recognition receptors (PRR), such as cGAS, TLR9 and NLRP3 inflammasome, in the innate immune system [2–4]. Additionally, both cytosolic and extracellular mitochondrial DNA (mtDNA) engage PRRs and trigger type I interferon (IFN) and interferon-stimulated gene (ISG) expression [5]. The regulations can be looped to a more elaborate network. For example, it has been recently reported that TLRs trigger IRF1-dependent transcription of CMPK2, a rate-limiting enzyme supplying deoxyribonucleotides for mtDNA synthesis. After exposure to NLRP3 activators, CMPK2-dependent mtDNA synthesis is required for producing cytosolic oxidized mtDNA fragments that associates with the NLRP3 inflammasome complex for the activation of NLRP3 inflammasome in macrophage [6].

Mitochondrial defects cause many diseases [7, 8]. Transcription factor A, mitochondrial (TFAM) is nuclear-encoded and a member of the high mobility group (HMG) proteins. It binds to mtDNA and regulates mtDNA replication and transcription, coordinating mitochondrial DNA assembly, regulating mtDNA copy number and mitochondrial functions [9–11]. Previous studies show that TFAM is associated with inflammatory diseases, such as neurodegenerative diseases [12–15] and asthma [16, 17]. Anti-inflammatory drug therapy has been considered as the first-line treatment in managing all grades of asthma severity [18].

In addition to inflammation, asthma is also characterized by bronchial hyperresponsiveness and remodeling [18]. The increased bronchial smooth muscle (BSM) mass is a feature of bronchial remodeling, which correlates with the decreased lung function [19, 20]. BSM cells in asthmatic patients showed an increased proliferation, correlating with an increased mitochondrial biogenesis and mitochondrial mass which are associated with an increased expression of peroxisome proliferator activated receptor g coactivator 1a (PGC-1a) and TFAM [20–22]. The involvement of calcium channel in the increased BSM mass in asthmatic patients has been affirmed [22, 23]. With the addition of a calcium channel blocker, the effects of the anti-inflammatory treatments in reducing the BSM thickness were significantly enhanced [23]. Nevertheless, the role of TFAM in asthma related inflammation remains obscure.

The mechanisms of TFAM in inflammation are emerging. Similar as its structural homology HMGB1, TFAM can act as a damage-associated molecular pattern molecule (DAMP), an endogenous intracellular protein released from damaged or dying cells, inducing pro-inflammatory and cytotoxic responses of microglia in vitro and regulating the expression of multiple mediators [24–27]. Nevertheless, TFAM more likely regulates inflammation via its function in regulating mtDNA packing and hemostasis. For example, Tfam deficiency in mice induces mtDNA stress, which primes the cytosolic antiviral innate immune response both in MEF (mouse embryonic fibroblasts) and bone marrow derived macrophages (BMDM). The cytosolic mtDNA engages cGAS and promotes STING-IRF3-depedent signaling to increase the expression of ISG [16]. In addition to its DNA binding activity, three independent RNA interactome studies based on cross-linking, oligo (dT) capture and MS analysis of the proteins associated with poly(A)-containing RNAs have revealed that TFAM is associated with RNA as well [28–30], it is unclear yet whether TFAM regulates any of the mRNA biogenesis and/or metabolic processes.

To explore the potential regulatory mechanisms of TFAM in asthma, we conducted the RNA-seq based on TFAM overexpression and control in vitro. As human lung epithelial cells are the first barrier in airways and are involved in many important functions in vivo, we employed A549 cells in this research, which have been widely used in asthma studies [31–35]. A549 cells have also been reported to have important roles in inflammation [36, 37]. Thus, we overexpressed TFAM in A549 cells to get the transcriptional profiles to research the mechanism in asthma in vitro. Transcriptome analysis showed that 642 and 169 genes were down-regulated and up-regulated, respectively, by the TFAM overexpression. The TFAM-repressed genes were highly enriched in cytokine-mediated signaling, interferon signaling pathways, immune and viral responses. We demonstrated that TFAM regulates thousands of alternative splicing events (ASEs), with the genes containing TFAM-regulated ASEs being enriched in transcription regulation. Further analysis showed that the promoters of TFAM-repressed genes were enriched in DNA binding motifs of transcription factors whose alternative splicing regulated by TFAM.

## Material and methods

### Plasmid construction, cell culture and transfection

The human TFAM gene was cloned by reverse transcription from total RNAs extracted from multiple cancer and non-cancer cell lines, followed by Polymerase Chain Reaction (PCR) amplification. The DNA fragment corresponding to a complete complementary DNA (cDNA) length was purified from a gel (MinElute PCR Purification Kit, Qiagen, 28004), and then cloned into the pIRES-hrGFP-1a vector (240031, Agilent Technologies) using the hot fusion method were designed with CE Design V1.04 (Vazyme Biotech Co., Ltd). Each of the primer comprises a fragment of gene specific sequence and a 17–30 bp sequence of the pIRES-hrGFP-1a vector (ABLife, Wuhan) [38, 39]. Please be noted that this vector also harbored a FLAG tag, which was fused to the 3’ end of TFAM. The primers used for cDNA cloning were as follow,

TFAM F Primer: agcccgggcggatccgaattcATGGCGTTTCTCCGAAGCA.

TFAM R Primer: gtcatccttgtagtcctcgagACACTCCTCAGCACCATATTTTCG.

The pIRES-hrGFP-1a vector was digested by EcoRI (NEB, 3101S) and XhoI (NEB R0146V) at 37 ℃ for 2 h ~ 3 h. Then enzyme-digested vector was run on 1.0% agarose gel and purified by Qiagen column kit (MinElute PCR Purification Kit, Qiagen, 28004). Then the insert fragment was synthesized by PCR amplification and PCR insert were added to a PCR microtube for ligation (T4 DNA ligase, NEB, M0202V) with ClonExpress® II One Step Cloning Kit (Vazyme, C112). Plasmids were introduced into Escherichia coli strain by chemical transformation. Cells were plated onto LB agar plates containing 1µL/ml ampicillin (sigma, 7177-48-2), and incubated overnight at 37 ℃. Colonies were screened by colony PCR (28 cycles) with universal primers (located on the backbone vector). The insert sequence was verified by Sanger sequencing.

Human lung epithelial cancer cell line A549 cells were obtained from CCTCC (China Center for Type Culture Collection, Wuhan, Hubei, China) with the catalog number GDC0063 in 2017. The cell line has been authenticated with STR analysis by Cell Bank, Type Culture Collection, Chinese Academy of Sciences (CBTCCCAS), and tested for the free of mycoplasma contamination. STR analysis was performed as previously described [40].

A549 cells were cultured in 24-well plates with 5% CO_2_ at 37 ℃ in Ham's F-12K (Kaighn's) medium containing 10% FBS (fetal bovine serum), 100 U/mL penicillin and 100 µg/mL streptomycin. Cells were cultured to a confluence of 50–60% and transfected by pIRES-hrGFP-1a vector containing the TFAM gene (TFAM-OE) or the empty vector (control) performed using Lipofectamine 2000 (Invitrogen, Carlsbad, CA, USA, 11668019) according to the manufacturer's protocol. Transfected cells were harvested by washing twice with 1 × PBS buffer, followed by collection using Trizol for total RNA preparation and using washing buffer containing 1% SDS and 2% PMSF for total protein extraction, when the culture was reached a confluence of 80–90% after 48 h post-transfection.

### Western blotting analysis

Total proteins were prepared from the collected cells after 5 min boiling in the loading buffer containing β-mercaptoethanol and SDS, followed by separation on a 12% SDS-PAGE gel and transferred onto 0.45 mm PVDF membranes. The PVDF membranes were then blocked and incubated overnight with primary antibody against FLAG tag (anti-FLAG, antibody produced in rabbit, sigma, F7425), followed by an incubation with horseradish peroxidase-conjugated secondary antibody, as previously described [39]. Then, membranes were visualized through chemiluminescence.

### RNA extraction, library construction and sequencing

The total RNA of A549 cells was extracted by TRIZOL (Ambion), and 1 µg of the total RNA was used for RNA-seq library preparation and a subsequent sequencing on Illumina HiSeq X Ten system (150 nt paired-end) [39].

### RNA-Seq raw data clean and alignment

Raw reads containing more than 2-N bases were first discarded. Then adaptors and low-quality bases were trimmed from raw sequencing reads using FASTX-Toolkit (Version 0.0.13). The short reads less than 16nt were also dropped. After that, clean reads were aligned to the GRch38 genome by tophat2 [41] allowing 4 mismatches. Uniquely mapped reads were used for gene reads number counting and FPKM calculation (fragments per kilobase of transcript per million fragments mapped) [42].

### Differentially expressed genes (DEG) analysis

The R Bioconductor package edgeR [43] was utilized to screen out the differentially expressed genes (DEGs). A false discovery rate < 0.05 and fold change > 2 or < 0.5 were set as the cut-off criteria for identifying DEGs.

### Alternative splicing analysis

The alternative splicing events (ASEs) and regulated alternative splicing events (RASEs) between the samples were defined and quantified by using the ABLas pipeline as described previously [44]. In brief, ABLas detection of ten types of ASEs was based on the splice junction reads, including exon skipping (ES), alternative 5' splice site (A5SS), alternative 3' splice site (A3SS), intron retention (IR), mutually exclusive exons (MXE), mutually exclusive 5'UTRs (5pMXE), mutually exclusive 3'UTRs (3pMXE), cassette exon, A3SS&ES and A5SS&ES. To assess Retinol-Binding Protein (RBP) regulated ASE, Student’s t-test was performed to evaluate the significance of the ratio alteration of AS events. Those events which were significant at *P*-value cutoff corresponding to a false discovery rate cutoff of 5% were considered RBP regulated ASEs.

### Reverse-transcription quantitative polymerase chain reaction (RT-qPCR)

The information of primers for validation of DEGs and RASEs is presented in Additional file 2: Table S1. To detect alternative isoforms, we used a boundary-spanning primer for the sequence encompassing the junction of constitutive exon and alternative exon as well as an opposing primer in a constitutive exon. The boundary-spanning primer of alternative exon was designed according to “model exon” to detect model splicing or “altered exon” to detect altered splicing. The same total RNA used for RNA-seq cDNA library preparation was used for qRT-PCR analysis. RNA was reverse transcribed into cDNA using an M-MLV Reverse Transcriptase (Vazyme). Real-time PCR was performed with the StepOne RealTime PCR System using the SYBR Green PCR Reagents Kit (Yeasen) as previously described [39]. For the purpose of DEG validation, the RNA expression levels of the tested genes were normalized against that of GAPDH.

### Functional enrichment analysis

To sort out functional categories of DEGs, Gene Ontology (GO) terms and Kyoto Encyclopedia of Genes and Genomes (KEGG) pathways were identified using KOBAS 2.0 serve [45]. Hypergeometric test and Benjamini Hochberg FDR controlling procedure were used to define the enrichment of each term.

### Prediction of the transcription factor binding sites in TFAM-repressed genes

HOMER [46] was used to identify the over-representative motifs (*P*-value, 0.01) in the promoter regions of TFAM repressed genes. The over-representative motifs and TFAM-regulated transcription factors (TFs) were paired by TFBSTools (http://www.bioconductor.org/packages/release/bioc/html/TFBSTools.html) that applied transcription factor database JASPAR2020 (http://jaspar.genereg.net/).

### Pearson’s correlation analysis

Pearson’s correlation (correlation efficient, 0.95; *P*-value, 0.005) was performed between the alternative splicing ratios of TFAM-regulated TFs and the expression levels of TFAM-repressed genes containing the TF binding motifs, to delineate their relationships. Cytoscape (v3.5.1) was used to depict the correlation network.

## Results

### TFAM overexpression preferentially down-regulates gene expression in A549 cells

**Fig. 1:**
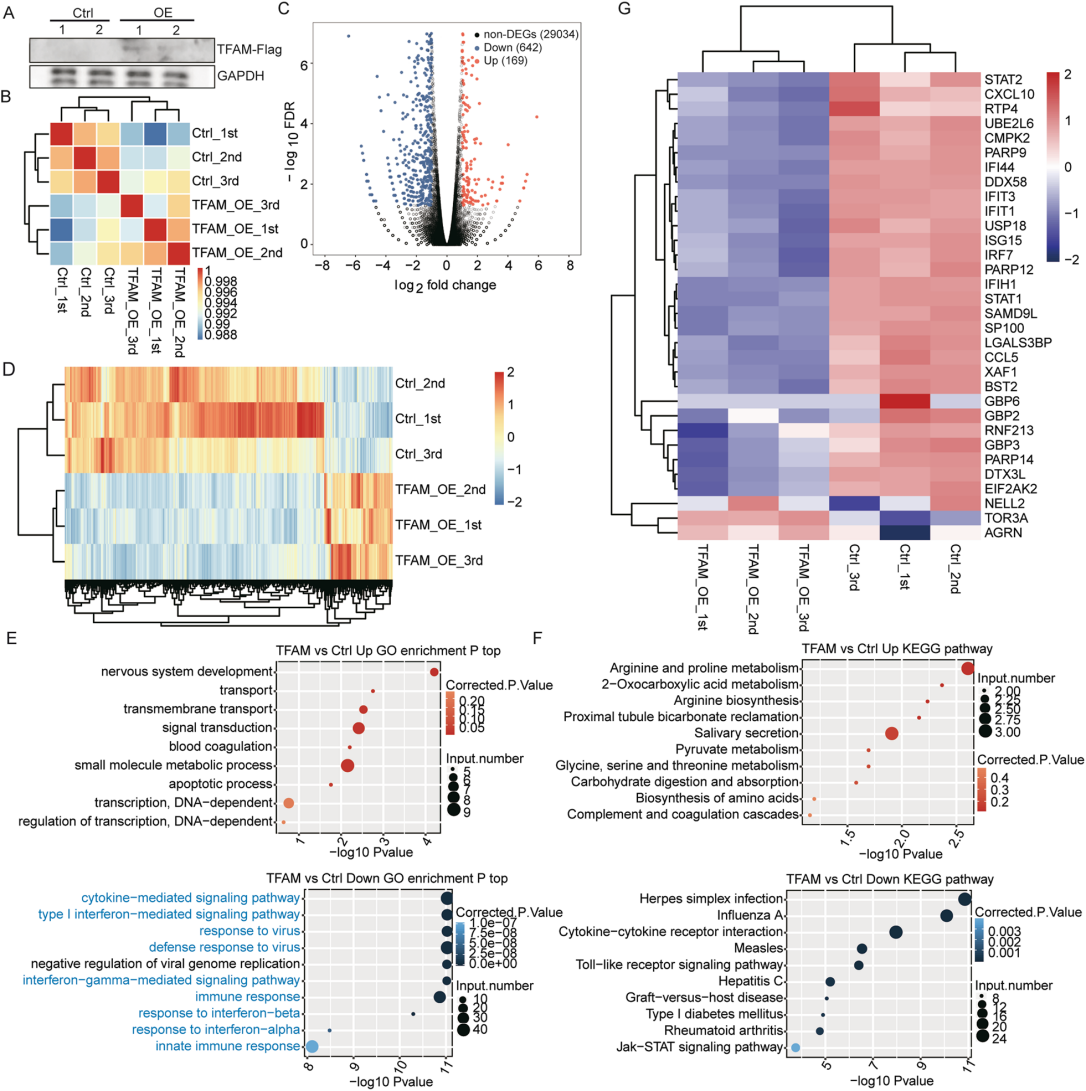
TFAM selectively represses gene expression in human lung epithelial A549 cells. **a** Enforced expression of TFAM in A549 cells detected by western blot experiment, gels and blots were cropped. **b** Heat map display of the hierarchically clustered Pearson’s correlation matrix resulting from comparing the expression level of each gene in the control and TFAM-overexpression transcriptomes. **c** Volcano plot shows the expression change of genes between the control and TFAM-overexpression transcriptomes, leading to the identified DEGs. Upregulated genes (FC ≥ 2; FDR < 0.05) are labeled in red and downregulated genes (FC ≤  − 2; FDR < 0.05) are labeled in blue. **d** Hierarchical clustering of the expression levels of all the identified DEGs in control and TFAM-overexpression samples. FPKM values are log2-transformed and then median-centered by each gene. **e** The top 10 GO biological processes of TFAM-up and down-regulated genes. **f** The top 10 KEGG pathways of TFAM-up and down-regulated genes. **g** Hierarchical clustering of the expression levels ISGs in the control and TFAM-overexpression A549 cells; these ISGs were negatively regulated in the Tfam-deficiency mouse murine embryonic fibroblast (MEF) cells [47]

We overexpressed human TFAM gene in human lung epithelial cancer cell line A549 to study TFAM-regulated transcriptome. A549 cells were transfected by TFAM overexpressing plasmid (TFAM-OE) and empty vector (control). The results we gained from the experiment have to be tested in order to prove their reliability. The success of TFAM overexpression was validated by western blot analysis using antibody against the FLAG tag (Fig. 1a). A total of six cDNA libraries were constructed and sequenced for the control and TFAM-OE in A549 cells, with three biological replicates for each group (Ctrl_1st, Ctrl_2nd, Ctrl_3rd, TFAM_1st, TFAM_2nd, TFAM_3rd). The raw reads from RNA-seq were cleaned, and the cleaned reads were then mapped to the human genome. The amount of the cleaned reads that were mapped to the human genome was averagely 81 million per sample and the amount of uniquely mapped reads averagely 75 million (Additional file 3: Table S2).

The uniquely mapped reads were used for the following analysis. The fragments per kilobase of transcript per million fragments mapped (FPKM) was used to present the level of gene expression. We obtained 24, 593 expressed genes when FPKM was set higher than 0 and 12,158 genes when FPKM >1 in at least one sample (Additional file 4: Table S3). On the base of the results of FPKM, a correlation of matrix was built up (Fig 1b). The correlation matrix showed a high similarity among all six transcriptome datasets, indicating the confidence of the experiments. Nevertheless, the three biological replicates in the same group of samples showed a high correlation than between two different groups (Fig 1b), indicating that increased expression of TFAM imposed a consistent transcription difference.

To explore the gene expression differences between TFAM-OE and control, a volcano map was drawn on the base of edgeR. The volcano map showed the up-regulated genes and down-regulated genes. A standard of fold change (FC) ≥ 2 or ≤ 0.5 and a 5% false discovery rate (FDR) was used to separate the significantly regulated genes from the other genes, resulting in 169 significantly up-regulated genes and 642 down-regulated genes (Fig. 1c). The details of the DEGswere presented in Additional file 5: Table S4. Moreover, heatmap plot of the FPKM values of all DEGs showed a clear separation between the TFAM-OE and control group, and a high consistency among three replicates of the same groups (Fig. 1d). All the results above supported a conclusion that TFAM shows a significantly higher capacity of down-regulating than up-regulating gene expression.

### TFAM-repressed genes are enriched in cytokine-mediated signaling, interferon response and signaling, viral and immune responses

To explore the potential biological functions of the TFAM-regulated genes, GO and KEGG were used to analyze the DEGs. There were 385 down-regulated and 102 up-regulated DEGs being annotated by GO, and 459 down-regulated and 120 up-regulated DEGs annotated by KEGG (Additional file 6: Table S5). The GO biological process mostly enriched by the up-regulated genes was nervous development, which might be related to TFAM function in neurological diseases (Fig. 1e, upper panel, Additional file 5: Table S4). The KEGG pathways enriched by TFAM-upregulated genes were mostly related to metabolisms (Fig. 1f, upper panel, Additional file 5: Table S4).

Strikingly, the TFAM down-regulated genes were highly enriched in anti-viral GO biological processes, which involved 9 of the top ten enriched processes including cytokine-mediated signaling pathway, type I interferon-mediated signaling pathway, response/defense response to virus, interferon-gamma-mediated signaling pathway, immune response, response to interferon-beta/alpha, and innate immune response (Fig. 1e, lower panel). Consistently, Herpes simplex infection, influenza A, measles, cytokine-cytokine receptor interaction and Toll-like receptor signaling pathway were the top five enriched KEGG pathways by TFAM down-regulated genes, which included three viruses and two viral response pathways. We noted that most of the interferon-stimulated genes (ISGs) that have previously shown an upregulated expression in Tfam deficient mouse MEF cells [16] demonstrated a marked increase in their expression in the TFAM-overexpressing A549 cells (Fig. 1g). This consistency indicates that TFAM functions in repressing antiviral responses are similar in human and mice.

**Fig. 2:**
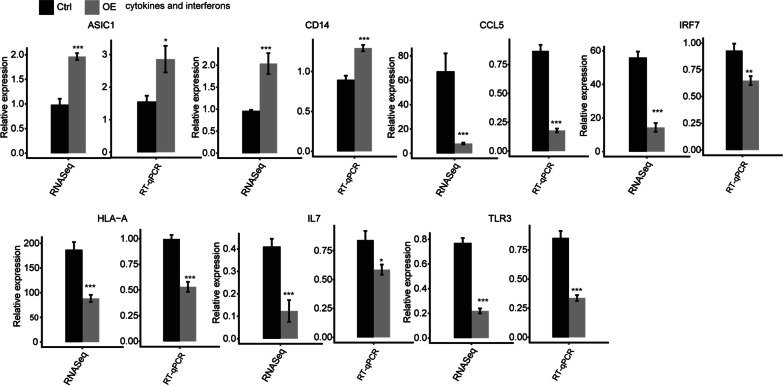
Validation of the TFAM-regulated gene expression in A549 cells. The expression levels of the two upregulated and five representative down-regulated genes were analyzed by RT-qPCR experiment. The quantification results from RNA sequencing data and RT-qPCR were showed in parallel. FPKM values were calculated as that has been explained in Materials and Methods. Error bars represent mean ± SEM. ***p < 0.001

In order to verify the regulation of TFAM regulated gene expression, 2 upregulated and 5 down-regulated DEGs were randomly selected and tested by RT-qPCR experiment (Fig. 2). The cDNA generated from the control and TFAM-OE transfected cells were used as template. Among these genes, several were classical inflammation and antiviral response genes, CCL5, IL7, IRF7 and TLR3. TFAM-regulated expression of all these 7 genes demonstrated a satisfied consistency by both RNA-seq and RT-qPCR experiments, supporting the reliability of the data of RNA-seq.

### TFAM regulates thousands of alternative splicing events, particularly repressing exon skipping

The RNA binding capability of TFAM encourages us to propose that TFAM may affect some of the mRNA biogenesis and/or metabolic processes [28–30]. To pursue this possibility, we analyzed the changes in alternative splicing events between the transcriptomes of the control and TFAM-OE A549 cells. The alternative splicing events (ASEs) were classified into nine categories, which includes an intron retention (IR) event, and 8 non-IR (not intron-retention) events including mutually exclusive 3’ UTR (3pMXE), mutually exclusive 5’ UTR (5pMXE), alternative 3’ splice site (A3SS), A3SS&exon skipping (ES), alternative 5’ splice site (A5SS), A5SS&ES, cassette exon, exon skipping (ES).

**Fig. 3:**
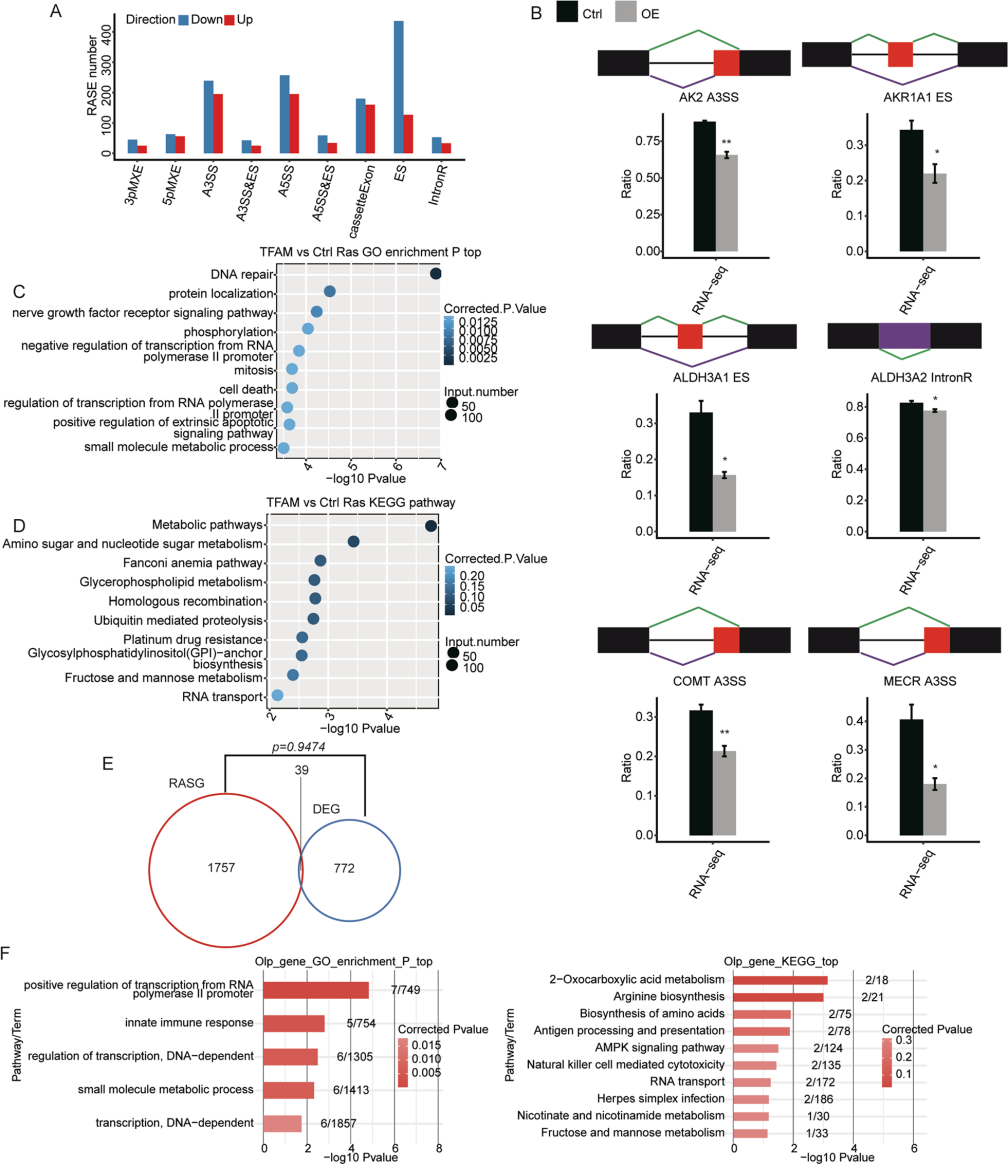
Identification and functional clustering of the TFAM-regulated alternative splicing events. **a** Classification of the TFAM-regulated alternative splicing events (RASEs). **b** Plots of several representative RASEs regulated by TFAM. The schematic diagrams depict the structures of ASEs. Alternative splicing (AS) junctions, purple line; model splicing (Model) junctions, green line. The exon sequences are denoted by boxes and intron sequences by the horizontal line (top panel). RNA-seq quantification validation of ASEs are shown in the bottom panel. Error bars represent mean ± SEM. *p < 0.05, **p < 0.01. The altered ratio of AS events in RNA-seq were calculated using the formula: AS junction reads/(AS junction reads + Model junction reads). Plots of the top 10 GO biological processes (**c**) and KEGG (**d**) enriched by TFAM-regulated RASGs. **e** Venn diagram shows the overlap between the TFAM-regulated DEGs and TFAM-regulated RASGs; at least one TFAM-regulated RASE was present in each RASG. **f** The GO biological processes and KEGG pathways enriched by genes regulated by TFAM at both the transcriptional and alternative splicing levels

A total of 87437 ASEs were identified in this study, involving 23810 known ASEs and 61663 novel ASEs (Additional file 7: Table S6). To identify the TFAM-regulated alternative splicing events (RASEs), the changes of the alternative splicing (AS) ratio between TFAM-OE and control transcriptomes were compared. By setting a cut-off of *P*-value 0.05, 2563 RASEs were identified, containing 331 IR and 2232 non-IR (NIR) RASEs (Fig. 3a, Additional file 8: Table S7). Several examples of the quantification results of the RASEs using RNA-seq junction reads were shown in Fig. 3b. These RASEs were primarily ES, CassetteExon, A3SS and A5SS (Fig. 3a). We noticed that when compared to the cassette exon events, TFAM overexpression resulted in more ASEs that demonstrated a decreased level of exon skipping. This suggested that TFAM may specifically represses exon skipping in an unknown mechanism.

### TFAM regulated alternative splicing events are enriched in DNA repair, metabolic pathways and transcriptional regulation

We assessed the potential function of TFAM-regulated alternative splicing by performing GO and KEGG analyses on regulated alternative splicing genes (RASGs). GO analysis showed that TFAM-regulated alternative splicing were mostly enriched in DNA repair pathway, followed by protein localization and nerve growth factor receptor signaling pathway (Fig. 3c). Metabolic pathways were most enriched by TFAM-regulated RASGs in the KEGG analysis results (Fig. 3d). Interestingly, we noticed that regulation and negative regulation of transcription from DNA polymerase II promoter were among the top 10 GO terms enriched by TFAM-regulated RASGs. These results suggested that TFAM may exert some of its biological functions via alternative splicing regulation.

When the 1796 RASGs were overlapped with 811 DEGs, and there were only 39 shared genes, showing no statistical significance (Fig. 3e). We therefore speculated that most of these genes might be regulated by TFAM independently at the transcription and alternative splicing levels. Interestingly, these 39 shared genes were strongly enriched in positive regulation of transcription from RNA polymerase II promoter (GO-biological process (BP)), which included DDIT3, IRF7, CSF3, IFI16, HELZ2, NLRC5, SMARCA4 (Fig. 3f, Additional file 9: Table S8). This indicated that transcription regulation genes may be coordinately regulated by TFAM at the transcription and alternative splicing levels.

**Fig. 4:**
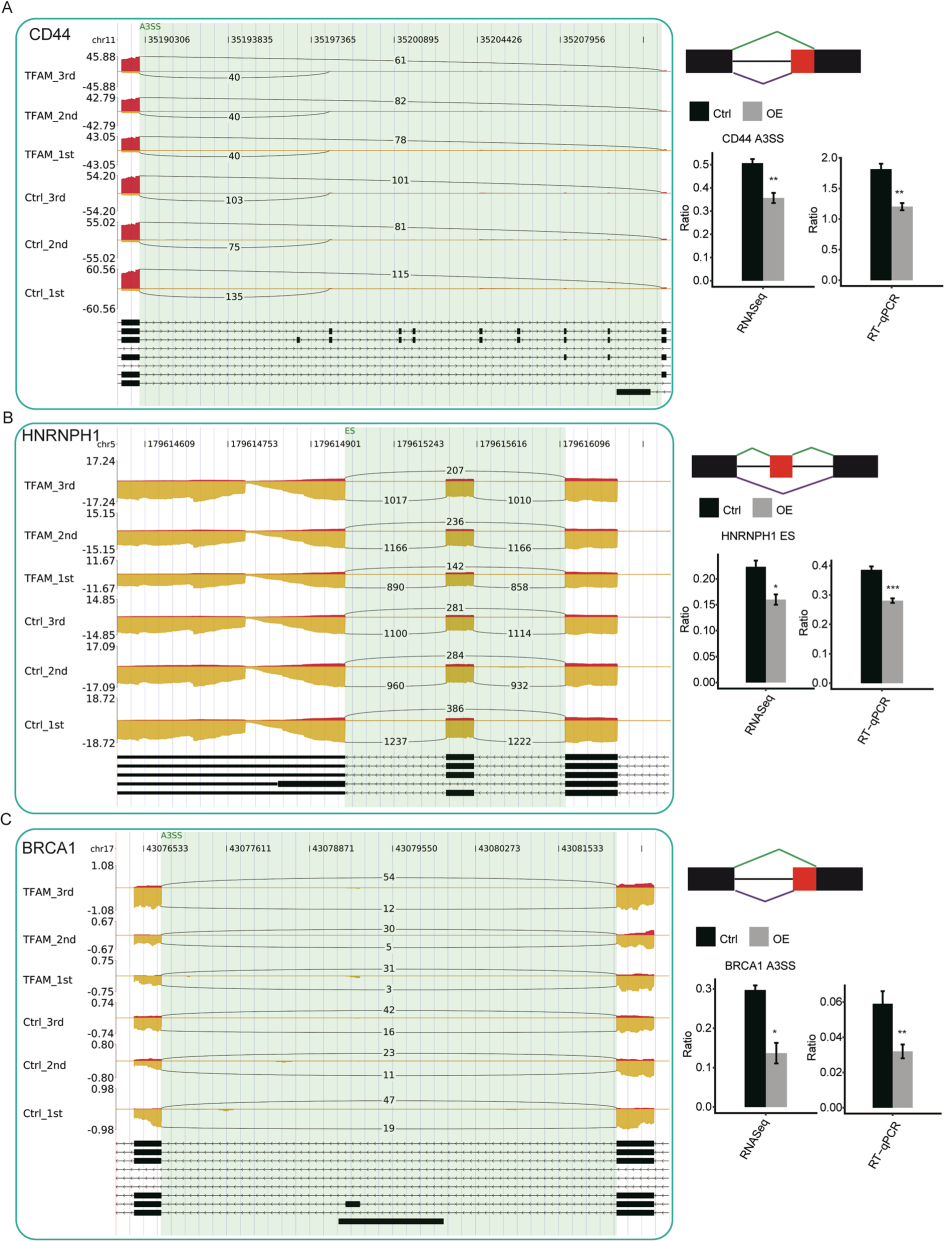
Validation of TFAM-regulated RASEs. The RNA-seq read distribution profiles of the RASEs subjected to RT-qPCR were depicted on the left, with the read density map and the numbers of alternative and model junction reads being shown. The black lines represent the splice junctions composing ASEs. The schematic diagrams depict the structures of ASEs are shown on the right upper panel, which are similar as in Fig. 3B. The results of the RNA-seq and RT-qPCR quantification of the change of the ratio between the alternative and model splicing products are shown on the right lower panel. Error bars represent mean ± SEM. *p < 0.05, **p < 0.01

To assess the confidence of the TFAM-regulated ASEs, six RASEs in DNA repair and cell apoptosis were randomly selected for performing RT-qPCR experiments. To enable a more clear comparison, we showed the RNA-seq quantification results, the RNA-seq reads distribution map with the number of the splicing junction reads labeled, and also the RT-qPCR results side by side (Fig. 4 and Additional file 1: Fig. S1). The exon skipping event in Among these tested RASEs, four were statistically consistent with the RNA-seq quantification results, which included CD44, HNRNPH1, BRCA1 and IKBKP, while those in HNRNPF and HNRNPR were not (Fig. 4 and Additional file 1: Fig. S1). We noticed that the inconsistency RASEs showed smaller change in the alternative splicing ratios upon the TFAM overexpression.

### TFAM-regulated alternative splicing is strongly linked to its repressed gene expression

**Fig. 5:**
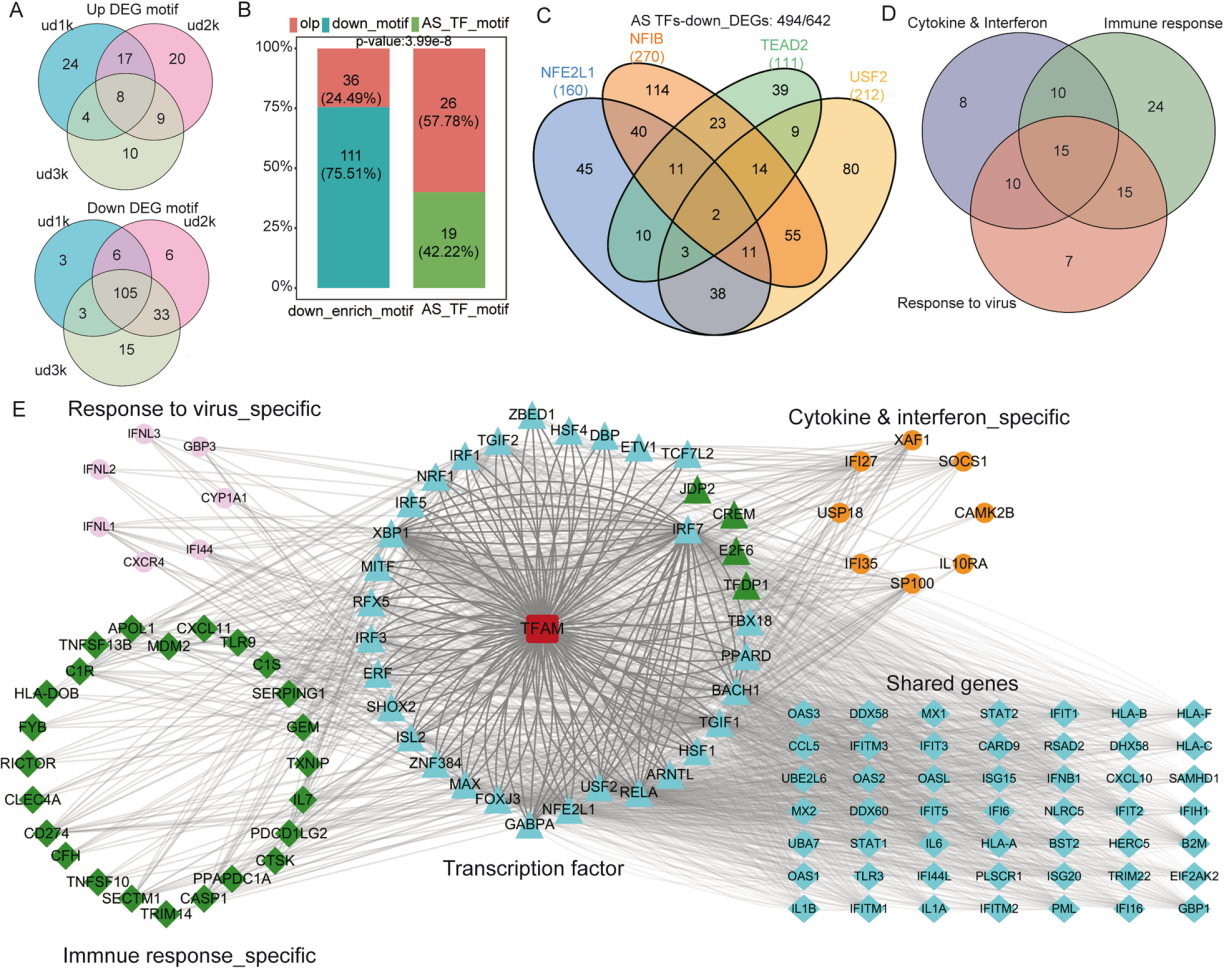
TFAM-regulated alternative splicing of the transcript factors are strongly linked to the TFAM-repressed gene expression. **a** Venn diagram shows the overlap of the DNA motifs enriched in the promoter regions of the TFAM-regulated genes. The overlap was performed among the motifs over-represented in the DNA regions that were 1 K, 2 K, and 3 K upstream and downstream from the transcription start sites. **b** Overlap of the DNA binding motifs of TFAM-regulated TFs (RASGs) with the DNA motifs enriched in the promoter regions of the TFAM-repressed genes (DEGs). **c** Venn diagram shows the overlap among the down-DEGs containing the DNA binding motifs for the top four TFs (NFIB, USF2, TEAD2, and NFE2L1) that were ranked by the number of down-DEGs harboring their DNA binding sites. **d** Overlap of genes that engage multiple functions. **e** Networks between the TFAM-regulated TFs and their targeted down-DEGs involved in the interferon/cytokine-mediated signaling pathways, immune/innate immune response, response/defense response to virus. The down-DEGs were grouped based on whether they are specific to each of the three classes of functions

Given that the genes containing TFAM-regulated ASEs were significantly enriched in regulation of transcription from DNA polymerase II promoter, we proposed that TFAM may regulate target gene transcription by affecting the alternative splicing of transcription factors. To test this hypothesis, we subjected all the RASE-containing genes to TFBSTools to identify transcription factors (TFs) with known binding motifs, which resulted in 45 such TFs (Additional file 10: Table S9A). A total of 683 transcription factors with their corresponding motif sequences were collected in TFBSTools. We then identified DNA motifs enriched in the promoter regions (1–3 K adjacent to the transcription start sites) of the TFAM-repressed genes and TFAM-promoted genes, demonstrating more consistently predicted motifs in the down-regulated genes than the upregulated genes (Fig. 5a, Additional file 10: Table S9B). We then ran TFBSTools to correspond the DNA binding motifs of TFs whose splicing was regulated by TFAM to the motifs enriched by TFAM-repressed and promoted genes separately. It was shown that the DNA binding motifs 26 of the 45 TFAM-regulated TFs (RASGs) were overlapped with 36 of the 147 motifs present in the promoters of TFAM-repressed genes (Fig. 5b); the overlap was remarkably significant (*P*-value 6.45e−9). One overlapped motif was usually present in many TFAM-repressed genes (Fig. 5c); 36 overlapped motifs were present in 638 of the 642 TFAM-repressed genes. As such, TFAM regulation of alternative splicing events in transcription factors has a potential to regulate the transcription of almost all of the TFAM-repressed genes.

We further analyzed the Pearson correlation between the TFAM-regulated alternative splicing and TFAM-regulated gene expression. Setting the cutoffs as correlation coefficient ≥ 0.95 and ≥ *P*-value 0.005, we found that the expression change of 602 TFAM-repressed genes were correlated with the alternative splicing ratio change of TFAM-regulated TFs. Among these 602 TFAM-repressed genes, we selected those involved in cytokine-mediated signaling (one GO-BP term), interferon signaling and response (4 GO-BP terms), response/defense response to virus (2 GO-BP terms), immune/innate immune response (2 GO-BP terms) (Fig. 1e, Additional file 1: Fig. S2C). These genes were integrated to three groups, many of which were shared (Fig. 5d). The correlation network between these selected TFAM-repressed genes and the corresponding TFAM-regulated TFs was drawn (Fig. 5e). The results further supported that TFAM-regulated alternative splicing may generally contribute to the TFAM-repressed expression of anti-viral response genes.

The connections between TFAM-regulated alternative splicing of transcription factors and TFAM promotion of gene expression were analyzed in parallel (Additional file 10: Table S9), showing a strong overlap between the DNA binding motifs of TFAM regulated TFs and motifs enriched by TFAM-upregulated genes (Additional file 1: Fig. S2A), and most of the TFAM-upregulated genes (transcription) containing DNA binding motifs of TFAM-regulated TFs (alternative splicing) (Additional file 1: Fig. S2B).

## Discussion

In previous reports, TFAM has been largely recognized as a mitochondrial transcription factor that is capable of binding mtDNA, regulating mitochondrial transcription and the mitochondrial DNA maintenance [48, 49]. Consistent with the recently emerging roles of mtDNA as a critical player in stimulating proinflammatory responses [47], reduced expression of Tfam in mouse embryonic fibroblast cells promotes antiviral innate immune response, which is attributed to the increased cytosolic mtDNA levels that engages the DNA sensor cGAS to promote the expression of interferon-stimulated gene (ISG), activate Type I interferon response and increase viral resistance [47]. In this study, overexpression of TFAM in A549 cells resulted in the selective decreased expression of ISGs, which is highly consistent with the results observed in mouse MEF cells. We demonstrated that TFAM overexpression selectively repressed the expression of genes in cytokine-mediated signaling pathway, both type I and type II interferon-mediated signaling pathways, response/defense response to virus, immune/innate immune response. These findings offer more comprehensive information in understanding the TFAM-regulated proinflammatory response in viral defense, and also in inflammation diseases such as asthma.

Asthma is primarily an inflammatory disorder of the airways [50]. TFAM has been reported to be participate in the asthma development by BSM [22], while functions of TFAM in lung epithelial cells unknown. As human lung epithelial cells was the first barrier in airways, A549 cells were used to detect the TFAM regulated transcriptome profile. We demonstrated that TFAM overexpression represses the expression of genes in cytokine-mediated signaling pathway and innate immune response, and toll-like receptor signaling pathway, suggesting that the increased TFAM might protect the lung epithelial cells from inflammation.

Asthma is also a metabolic disorder. Metabolisms of nitric oxide, vitamin D and arginine are deregulated [51–54]. Expression of nitric oxide synthase (iNOS) and arginase (ARG), arginine synthetic enzymes, and mitochondrial respiratory complexes III and IV are higher in asthmatic lung samples when compared with healthy controls [54, 55]. Arginase plays a key role in the pathogenesis of asthma and other pulmonary diseases by regulating L-arginine metabolism and NO homeostasis [56]. We demonstrated that the expression of several genes involved in arginine metabolism and biosynthesis was selectively elevated by TFAM overexpression, suggesting that TFAM may regulates arginine metabolism/biosynthesis. This interplay between TFAM and arginine metabolism may coordinately contribute to the pathology of asthma.

The current studies has been focused on the mitochondrial function of TFAM, it is unclear whether TFAM has any function in nucleus. In light of the recently reported RNA binding activity of TFAM [28–30], we have analyzed the TFAM-regulated transcriptome, leading to the findings that TFAM regulates over 2000 alternative splicing events, and preferentially inhibits the exon skipping events. Moreover, TFAM-regulated ASEs are enriched in regulation of transcription of RNA regulation from RNA polymerase II promoter, which includes key transcription factors regulating inflammation and innate immune responses, such as IRF7, IRF1 and IRF3 [57–60]. Our further studies have revealed that the DNA binding motifs of the TFAM-regulated transcription factors at the alternative splicing level are enriched in the TFAM-regulated DEGs. In fact, most of the TFAM-repressed DEGs harbors at least one DNA binding motif of the TFAM-regulated TFs at the alternative splicing level. As such, we proposed that TFAM regulation of the alternative splicing of TFs in nucleus may also contribute to its repressed expression of antiviral signaling, inflammation and immune response genes.

Among the genes regulated by TFAM at the alternative splicing levels, some have been reported to be associated with asthma. For example. An ALDH3A2 gene mutation was reported in accumulation of leukotriene B4, which is a key molecule and a pro-inflammatory mediator in asthma [61]. ALDH3A1 was reported as a potential biomarker for precise diagnosis of difficult-to-control asthma [62]. The putative association between allergic rhinitis and asthma with AKR1B1 variants was reported [63].

In the future studies, we wish to study how TFAM regulation of the alternative splicing of key transcription factors in nucleus contribute to the TFAM-regulated transcriptional regulation, and how TFAM-regulated transcription and alternative splicing in lung epithelial cells are associated with the bronchial smooth muscle mass increase and inflammation. The function of TFAM as an mtDNA regulator has been studied for many years, the finding of TFAM regulation of nuclear pre-mRNA splicing shall promote a more comprehensive recognition of TFAM regulation in human cells and various diseases.

## Supplementary Information

**Additional file 1**. **Fig. S1.** Validation of TFAM-regulated RASEs. The experiments and presentation of the results are the same as shown in Fig. 4. **Fig. S2.** TFAM-regulated alternative splicing of the transcript factors are linked to the TFAM-regulated gene expression. (**A**) Overlap of the DNA binding motifs of TFAM-regulated TFs (RASGs) with the DNA motifs enriched in the promoter regions of the TFAM-upregulated genes (DEGs). (**B**) Venn diagram shows the overlap among the up-DEGs containing the DNA binding motifs for the top four TFs (NFIB, USF2, TBX18, and E2F6 that were ranked by the number of up-DEGs harboring their DNA binding sites. (**C**) UpSet plot of the overlaps of the TFAM-repressed genes enriched in nine top GO-BP terms. The terms were grouped to four functional groups. The interferon function includes “type I interferon-mediated signaling pathway”, “interferon-gamma-mediated signaling pathway”, “response to interferon-alpha”, and “response to interferon-beta”. The cytokine function includes one term “cytokine-mediated signaling”. The response to virus function includes 2 terms, “response to virus” and “defense response to virus”. The immune function includes “immune response” and “innate immune response”.

**Additional file 2**. Primers of the genes validated by RT-qPCR.

**Additional file 3**. Summary of sample names, description, the RNA-seq sequencing information and mapping results in each sample.

**Additional file 4**. Gene expression level (FPKM).

**Additional file 5**. Differential expression of genes between TFAM-OE and control.

**Additional file 6**. List of GO terms and KEGG pathways enriched by DEGs.

**Additional file 7**. Statistics and classification of known and novel alternative splcing events.

**Additional file 8**. List of RASEs.

**Additional file 9**. The GO enrichment analysis of the genes overlapped by DEG and AS.

**Additional file 10**. The motif analysis.

## Data Availability

The data that support the findings of this study are available from the NCBI repository (GSE162384).

## Abbreviations

TFAM: Mitochondrial transcription factor A
DEGs: Differentially expressed genes
INF: Interferon
ASGs: Alternative splicing genes
ATP: Adenosine triphosphate
PRR: Pattern-recognition receptors
mtDNA: Mitochondrial DNA
ISG: Interferon-stimulated gene
HMG: High mobility group
BSM: Bronchial smooth muscle
DAMP: Damage-associated molecular pattern molecule
BMDM: Bone marrow derived macrophages
ASEs: Alternative splicing events
PCR: Polymerase chain reaction
cDNA: Complementary DNA
RT-qPCR: Reverse-transcription quantitative polymerase chain reaction
GO: Gene ontology
KEGG: Kyoto Encyclopedia of Genes and Genomes
FC: Fold change
FDR: False discovery rate
FPKM: Fragments per Kilobase of exon model per Million mapped fragments
MEF: Murine embryonic fibroblast
IR: Intron retention
non-IR: Not intron-retention
3pMXE: Mutually exclusive 3’ UTR
5pMXE: Mutually exclusive 5’ UTR
A3SS: Alternative 3’ splice site
ES: Exon skipping
A5SS: Alternative 5’ splice site
RASEs: Regulated alternative splicing events
AS: Alternative splicing
RASGs: Regulated alternative splicing genes
TFs: Transcription factors
BP: Biological process
