# Supplementary material for: Repression of the expression of proinflammatory genes by mitochondrial transcription factor A is linked to its alternative splicing regulation in human lung epithelial cells
Source: BMC Immunol. 2021 Dec 7;22:74. doi: 10.1186/s12865-021-00464-2 (PMC8650232; doi:10.1186/s12865-021-00464-2)
Supplement: Supplementary file 1 — Additional file 1. Fig. S1. Validation of TFAM-regulated RASEs. The experiments and presentation of the results are the same as shown in Fig. 4. Fig. S2. TFAM-regulated alternative splicing of the transcript factors are linked to the TFAM-regulated gene expression. (A) Overlap of the DNA binding motifs of TFAM-regulated TFs (RASGs) with the DNA motifs enriched in the promoter regions of the TFAM-upregulated genes (DEGs). (B) Venn diagram shows the overlap among the up-DEGs containing the DNA binding motifs for the top four TFs (NFIB, USF2, TBX18, and E2F6 that were ranked by the number of up-DEGs harboring their DNA binding sites. (C) UpSet plot of the overlaps of the TFAM-repressed genes enriched in nine top GO-BP terms. The terms were grouped to four functional groups. The interferon function includes “type I interferon-mediated signaling pathway”, “interferon-gamma-mediated signaling pathway”, “response to interferon-alpha”, and “response to interferon-beta”. The cytokine function includes one term “cytokine-mediated signaling”. The response to virus function includes 2 terms, “response to virus” and “defense response to virus”. The immune function includes “immune response” and “innate immune response”. [file 12865_2021_464_MOESM1_ESM.docx]

**Repression of the** **expression of proinflammatory genes by mitochondrial transcription factor A is linked to its alternative splicing regulation in human lung epithelial cells**

Jinsong Luo^1,a,#^, Hong Liu^1,a^, Daniel K. Jun Li^2,3^, Bin Song^2^, Yi Zhang^2^

1. Department of Pediatrics, Renmin Hospital of Wuhan University, Wuhan, Hubei, China
2. ABLife BioBigData Institute, Wuhan, Hubei, China
3. Department of Biology and Biotechnology, School of Chemistry, Chemical Engineering and Life Science, Wuhan University of Technology, Wuhan, Hubei, China

a. These persons contributed equally to this work.

^#^Correspondence: [1661617514@qq.com](mailto:1661617514@qq.com)


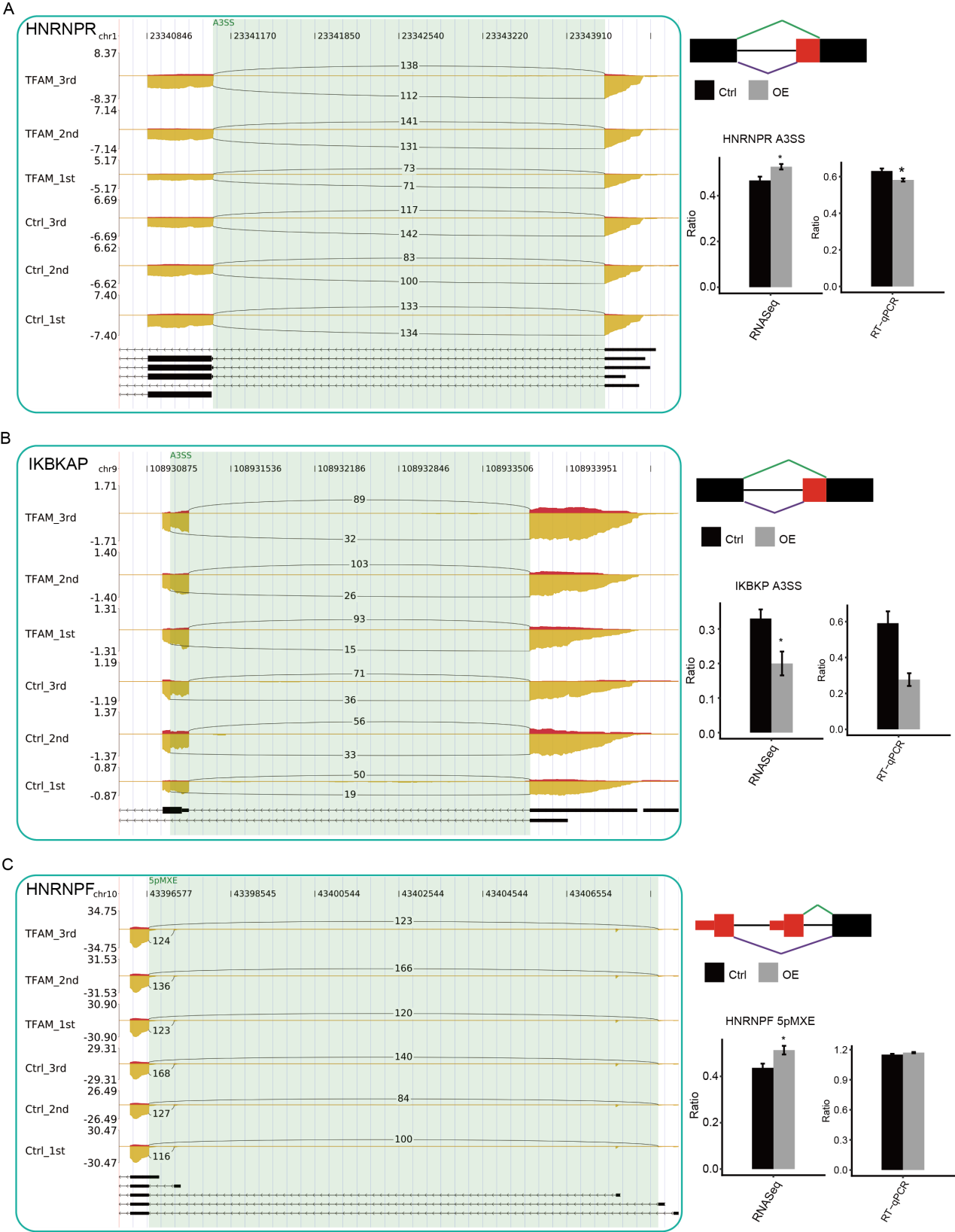


**Supplementary Fig. S1 Validation of TFAM-regulated RASEs.**

The experiments and presentation of the results are the same as shown in Fig. 4.


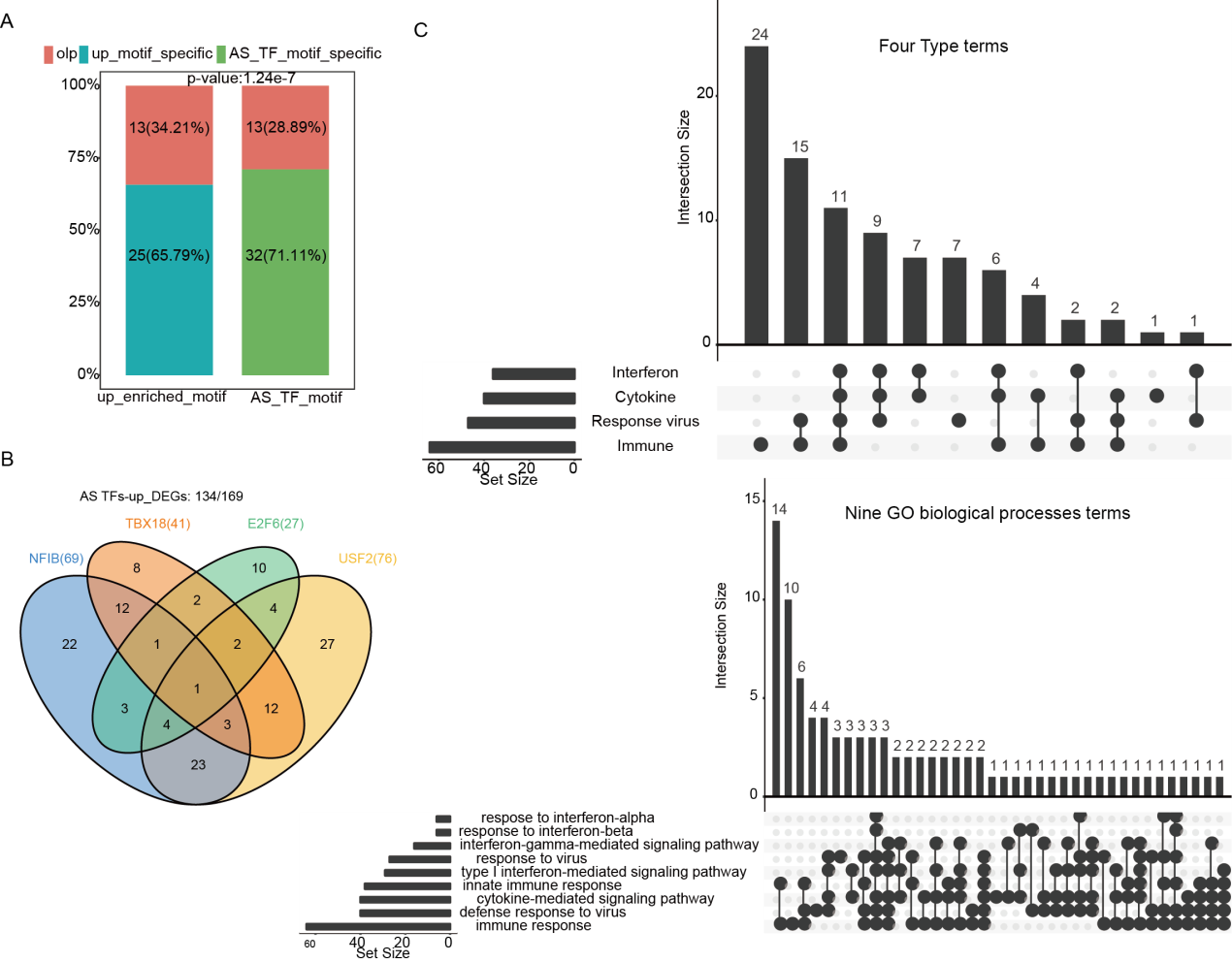


**Supplementary Fig. S2 TFAM-regulated alternative splicing of the transcript factors are linked to the TFAM-regulated gene expression.**

(A) Overlap of the DNA binding motifs of TFAM-regulated TFs (RASGs) with the DNA motifs enriched in the promoter regions of the TFAM-upregulated genes (DEGs). (B) Venn diagram shows the overlap among the up-DEGs containing the DNA binding motifs for the top four TFs (NFIB, USF2, TBX18, and E2F6 that were ranked by the number of up-DEGs harboring their DNA binding sites. (C) UpSet plot of the overlaps of the TFAM-repressed genes enriched in nine top GO-BP terms. The terms were grouped to four functional groups. The interferon function includes “type I interferon-mediated signaling pathway”, “interferon-gamma-mediated signaling pathway”, “response to interferon-alpha”, and “response to interferon-beta”. The cytokine function includes one term “cytokine-mediated signaling”. The response to virus function includes 2 terms, “response to virus” and “defense response to virus”. The immune function includes “immune response” and “innate immune response”.
